# Supplementary material for: Over Two-Fold Photoluminescence Enhancement from Single-Walled Carbon Nanotubes Induced by Oxygen Doping
Source: Nanomaterials (Basel). 2023 May 6;13(9):1561. doi: 10.3390/nano13091561 (PMC10180646; doi:10.3390/nano13091561)
Supplement: Supplementary file 1 [file nanomaterials-13-01561-s001.zip › nanomaterials-2366448-supplementary.pdf]

Over two-fold photoluminescence enhancement from single-walled carbon nanotubes induced by oxygen doping

Timofei Eremin, Valentina Eremina, Yuri Svirko and Petr Obraztsov

Department of Physics and Mathematics, University of Eastern Finland, Yliopistokatu 2, Joensuu 80101, Finland

SUPPLEMENTARY MATERIALS

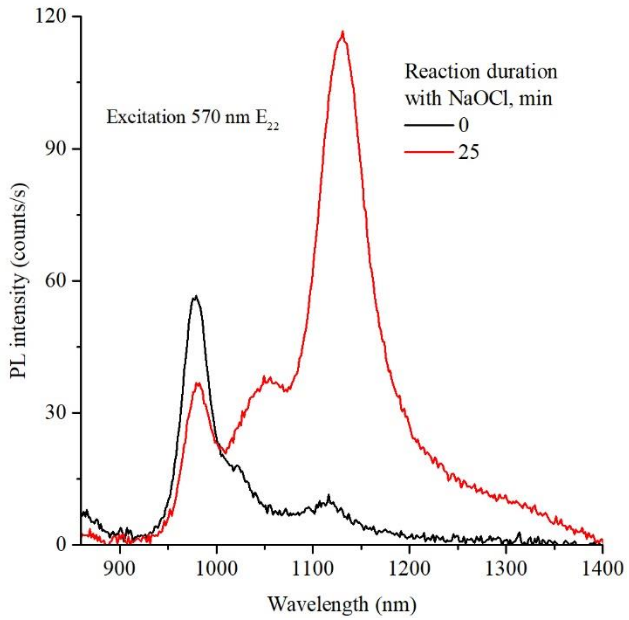

Fig. S1. PL spectra of pristine and doped SWCNT suspension. Excitation wavelength is 570 nm (horizontal cuts at 570 nm of Fig.1a and Fig1b of the main text)

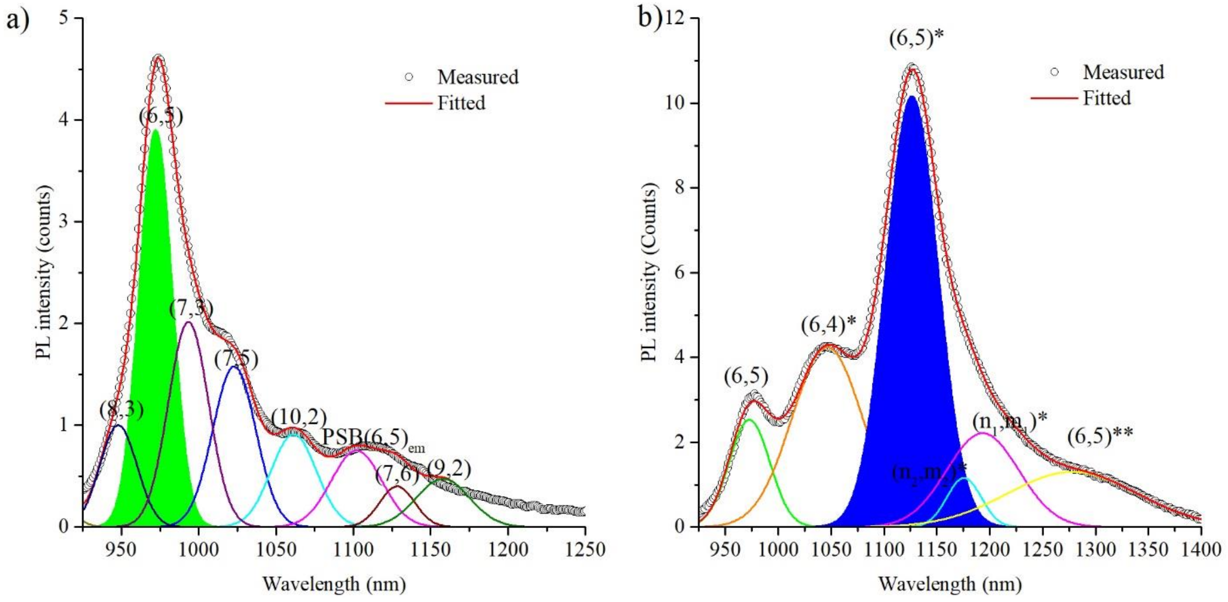

Fig S2. Deconvolution of PL spectra of SWNT suspension before (a) and after (b) oxygen doping procedure. (6,5)* peak corresponds to ether-d configuration of oxygen atom bonding, (6,5)** corresponds to epoxide-I configuration [18]. (n_1,m_1)* and (n_2,m_2)* corresponds to oxygen induced PL peak of SWNTs with unknown geometry persist in the sample.

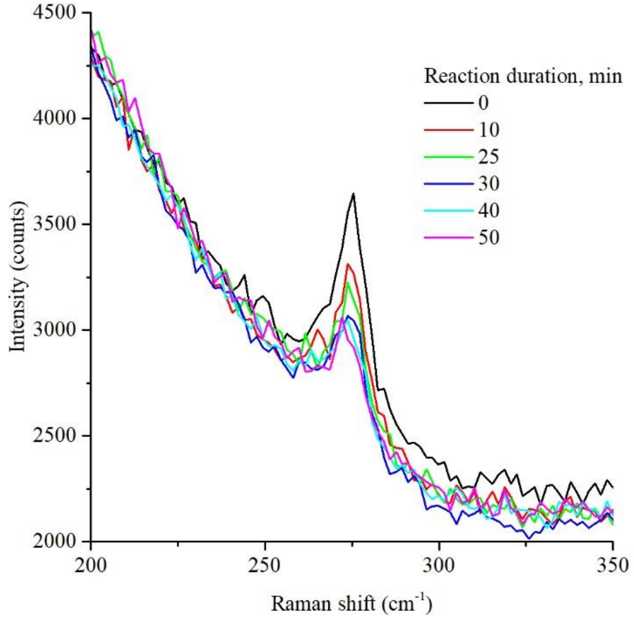

Fig S3. Raman spectra of pristine and doped SWCNT with different duration of doping procedure in RBM spectral region. Single peak at 270 cm^{-1} is radial breathing mode (RBM) peak.

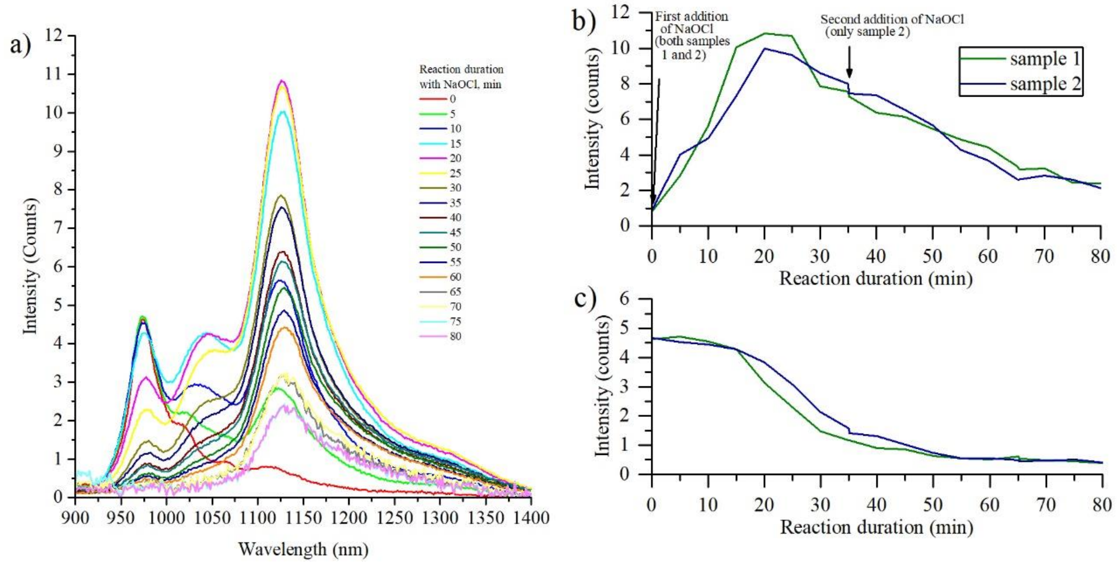

Fig. S4 Control experiment testing consumption of NaOCl as a limiting factor of PL intensity growth. a) Photoluminescence spectra of SWNT suspension with added NaOCl and irradiated with UV lamp for an indicated period. b,c) Dependency of (6,5)* (и) and (6,5) (c) PL peak intensities on the duration of UV exposure. An additional amount of NaOCl was added to sample 2 after 35 minutes of reaction duration
